# Association between *interleukin-18* gene polymorphism and *Helicobacter pylori* infection in the Korean population

**DOI:** 10.1038/srep11535

**Published:** 2015-06-22

**Authors:** Dae-Seong Myung, Wan-Sik Lee, Young-Lan Park, Nuri Kim, Hyung-Hoon Oh, Mi-Young Kim, Chan-Young Oak, Cho-Yun Chung, Hyung-Chul Park, Jong-Sun Kim, Sung-Bum Cho, Sun-Seog Kweon, Young-Eun Joo

**Affiliations:** 1 Department of Internal Medicine, Chonnam National University Medical School, Gwangju, Korea; 2 Department of Preventive Medicine, Chonnam National University Medical School, Gwangju, Korea

## Abstract

*Interleukin-18* (*IL-18*) is a pleiotropic, pro-inflammatory cytokine that is capable of promoting the Th1 response. A predominant Th1 response induces chronic and persistent inflammatory changes in the gastric mucosa in response to *Helicobacter pylori* (*H. pylori*) infection. The aim of this study was to investigate the potential association between *IL-18* gene polymorphisms and susceptibility to *H. pylori* infection in the Korean population. A total of 678 subjects who underwent a routine health check-up were enrolled. The *IL-18* gene polymorphisms at positions −656, −607, −137, +113, and +127 were genotyped. *H. pylori* positivity was demonstrated in 456 subjects (67.3%). The allele frequencies of *IL-18* gene polymorphisms at position −137 (rs187238) were different based on the status of *H. pylori* infection (G vs. C, adjusted OR 0.64 CI: 0.47–0.87, P = 0.005). The results indicate that the genetic variants in the *IL-18* gene may be associated with susceptibility to *H. pylori* infection in the Korean population, suggesting that IL-18 plays a role in the pathogenesis of *H. pylori*-associated diseases. However, this finding requires further replication and validation.

*Helicobacter pylori* (*H. pylori*) is a microaerophilic, Gram-negative flagellate bacterium that is trophic for the gastric epithelium. Most infections are asymptomatic, and once acquired, *H. pylori* can persist for prolonged periods, even decades. It is considered to be a major pathogen in gastritis, peptic ulcer, gastric adenocarcinoma, and gastric mucosa-associated lymphoid tissue (MALT) lymphoma^1^. Although the precise mechanisms and the pathogenic processes leading to *H. pylori*-associated diseases are not completely understood, recent evidence strongly suggests that these disorders are mediated by activated immune responses and that the inflammatory processes are influenced by environmental factors as well as host genetic factors^2^.

*H. pylori* infection triggers the downstream signaling events that result in the production of pro-inflammatory cytokines and other related genes in the gastric mucosa, shifting immunity towards the T-helper1 (Th1) response^3^,^4^,^5^,^6^. Therefore, genetic polymorphisms of the pro-inflammatory cytokines and other related genes that are involved in inflammatory and immune responses may influence persistent *H. pylori* infection and clinical outcomes in *H. pylori*-associated diseases.

Genetic factors that confer susceptibility to *H. pylori* infection have been extensively studied, and single-nucleotide polymorphisms (SNPs) of candidate genes involved in the inflammatory and immune response, including IL-1A, IL-1B, IL-1RN, IL-8, IL-10, myeloperoxidase (MPO), tumor necrosis factor-α (TNF-α) and TNF-β, have been examined^7^,^8^,^9^. These data suggest that the genetic backgrounds of *H. pylori* infection are somewhat different.

IL-18 is now recognized as an important regulator of innate and acquired immune responses, including potent stimulation of interferon (IFN)-γ production, enhancement of cell cytotoxicity of natural killer cells, and stimulation of Th1 cell differentiation^10^,^11^,^12^; it plays a critical role in chronic inflammation, in autoimmune diseases, and in a number of infectious diseases^13^,^14^,^15^,^16^,^17^,^18^,^19^. The human *IL-18* gene is located on chromosome 11q22.2–q22.3, and the production and activity of IL-18 is regulated by the *IL-18* promoter gene^20^,^21^,^22^. Accordingly, polymorphisms in the *IL-18* promoter gene are attractive candidates due to their impact on pro-inflammatory cytokine production and subsequently on inter-individual disease susceptibility. The existence of SNPs was investigated at the *IL-18* promoter gene, and five SNPs (−656G/T, −607C/A, −137G/C, +113T/G and +127C/T) were recognized^20^,^21^,^22^. These polymorphisms in the *IL-18* promoter gene have been noted as being associated with various inflammatory diseases, such as inflammatory bowel disease, rheumatoid arthritis, allergic diseases, and asthma^23^,^24^,^25^,^26^.

Recently, elevated *IL-18* mRNA and protein levels were observed in *H. pylori*-infected patients as compared with *H. pylori*-uninfected individuals^27^,^28^,^29^,^30^,^31^,^32^,^33^,^34^. IL-18 also has an important function in antimicrobial treatment response^33^,^34^, and a recent murine study using IL-18 knockout mice has shown that IL-18 is essential for the development of protective vaccine-induced immunity against *H. pylori* infection (35). These findings suggest that *IL-18* may influence the host response to *H. pylori* infection and may play multiple roles in *H. pylori*-associated diseases. However, to date, the association of *IL-18* gene polymorphisms and susceptibility to *H. pylori* infection has not been clarified. The aim of this study was to investigate the potential association between *IL-18* gene polymorphisms and susceptibility to *H. pylori* infection in the Korean population.

## Results

### Demographic and clinical characteristics of the study population

A total of 678 subjects were included in the study. Table 1 describes the demographic and clinical characteristics of the enrolled subjects, including age, sex, mean body mass index (BMI), smoking, alcohol, education, income level, *H. pylori* status, and relatives of gastric cancer. This study group comprised 402 males (59.3%) and 276 females (40.7%). The mean age was 53.9 ± 10.8 years with a range from 20 to 89 years. The BMI was 24.1 ± 3.0 with a range from 14.5 to 36.8. *H. pylori* positivity was demonstrated in 456 subjects (67.3%). Table 2 shows comparisons between the *H. pylori*-negative and -positive groups in demographic and clinical characteristics. There were no significant differences in the demographic and clinical characteristics between the 2 groups.

### Genotype and allele frequencies of *IL-18* gene polymorphisms in the *H. pylori*-negative and -positive groups

To investigate the relationship between the SNPs of the *IL-18* gene and susceptibility to *H. pylori* infection in the Korean population, *IL-18* gene polymorphisms at the −656, −607, −137, +113, and +127 position sites were genotyped using polymerase chain reaction based-restriction fragment length polymorphism (PCR-RFLP). No significant departure from Hardy–Weinberg equilibrium was observed for the five SNPs of *IL-18* gene in the *H. pylori*-negative group, suggesting that the genotypes and alleles were appropriately assigned. The genotype and allele frequencies of five *IL-18* SNPs in the *H. pylori*-negative and -positive groups were analyzed and are shown in Table 3. The *H. pylori*-positive group exhibited a higher GG genotype frequency at position −137 compared to the *H. pylori*-negative group. Compared with the GG genotype, the GC genotype was associated with a decreased risk of *H. pylori* positivity (OR 0.64, 95% CI: 0.45–0.92; aOR 0.64, 95% CI: 0.45–0.91). The frequencies of the TT and TG genotypes at position +113 (rs360718) were 68.9 and 28.4%, respectively, in the *H. pylori*-negative group and 77.4 and 21.5% in the *H. pylori*-positive group. The *H. pylori*-positive group exhibited a higher TT genotype frequency at position +113 than the *H. pylori*-negative group. Compared with the TT genotype, the TG genotype was associated with a decreased risk of *H. pylori* positivity (OR 0.67, 95% CI: 0.47–0.98; aOR 0.67, 95% CI: 0.46–0.97). The frequencies of the CC and TC genotypes at position +127 (rs360717) were 68.9 and 28.4% in the *H. pylori*-negative group, respectively, and 77.4 and 21.5% in the *H. pylori*-positive group. The *H. pylori*-positive group exhibited a higher CC genotype frequency at the position +127, than the *H. pylori*-negative group. Compared with the CC genotype, the TC genotype was associated with a decreased risk of *H. pylori* positivity (OR 0.67, 95% CI: 0.46–0.97; aOR 0.66, 95% CI: 0.46–0.96). The frequencies of the GG and GC genotypes at position −137 (rs187238) were 65.3 and 32.0%, respectively, in the *H. pylori*-negative group and 75.2 and 23.7% in the *H. pylori*-positive group. The C allele at position *−*137, G allele at position +113, and T allele at position +127 were less frequent in the *H. pylori*-positive group than in the *H. pylori*-negative group (18.7 *vs*. 12.9%, OR 0.65, 95% CI: 0.48–0.88; aOR 0.64, 95% CI: 0.47–0.87; 16.9 *vs*. 11.8%, OR 0.66, 95% CI: 0.48–0.90; aOR 0.66, 95% CI: 0.48–0.90; and 16.9 *vs*. 12.0%, OR 0.67, 95% CI: 0.49–0.92; aOR 0.66, 95% CI: 0.48–0.91, respectively). After adjustment using the Bonferroni correction method (assuming 9 independent tests), only the C allele at position −137 maintained the significance with a corrected p-value less than 0.05. However, no significant differences in the genotype and allele frequencies at positions −656 (rs1946519) and −607 (rs1946518) between the *H. pylori*-negative and -positive groups were found before Bonferroni correction.

### Haplotype frequencies of *IL-18* gene polymorphism in the *H. pylori*-negative and -positive groups

A complete linkage disequilibrium was found for the two polymorphisms at positions −656 (rs1946519) and −607 (rs1946518) as well as for the three polymorphisms at positions −137 (rs187238), +113 (rs360718), and +127 (rs360717) (R^2^ = 1, P < 0.0001). To evaluate whether certain *IL-18* haplotypes were associated with susceptibility to *H. pylori* infection, we analyzed the significant differences in haplotype frequencies between the *H. pylori*-negative and -positive groups, using HAPSTAT 3.0 software (University of North Carolina at Chapel Hill, Chapel Hill, North Carolina). Three haplotypes (GTC, CTC, and CGT) with frequencies > 0.002 were reconstructed. Table 4 shows the haplotype distributions of the *IL-18* gene in the *H. pylori*-negative and -positive groups. The most frequent haplotype in both the *H. pylori*-negative and -positive groups was GTC (81.1 *vs*. 86.8%). Compared with the most frequent haplotype GTC, the haplotype CGT (−137C/ +113G/ +127T) appeared to be a protective haplotype, with a frequency of 16.7% in the *H. pylori*-negative group and 11.6% in the *H. pylori*-positive group (aOR 0.64, 95% CI: 0.46–0.88, P = 0.006). However, no significant association was detected between the other haplotypes and risk of *H. pylori* infection.

## Discussion

The main immunologic response in gastric mucosa induced by *H. pylori* infection is characterized by the production of many pro-inflammatory cytokines associated with the development of *H. pylori*-associated diseases^1^,^2^,^3^,^4^,^5^,^6^. *IL-18*, produced mainly by local activated monocytes/macrophages, is a key pro-inflammatory cytokine that is observed in many aspects of the development of inflammation and Th1 responses^10^,^11^,^12^ and is increased in *H. pylori* infection^27^,^28^,^29^,^30^,^31^,^32^,^33^,^34^.

The prevalence of *H. pylori* infection varies among countries. *H. pylori*-associated inflammation and diseases vary in degree and type, depending on the regions with high or low prevalence of *H. pylori* infection^1^,^2^. Therefore, both genetic and environmental factors may contribute to the host susceptibility and outcome of *H. pylori* infection. *H. pylori* infection is influenced by interactions of multiple susceptibility genes and environmental factors, and no single gene or environmental factor exerts a large effect on susceptibility to *H. pylori* infection.

Previously, *IL-18* transcript and protein levels increased in the gastric mucosa of *H. pylori*-infected patients and decreased dramatically after *H. pylori* eradication. The strong correlation between gastric mucosal IL-18 levels and inflammatory cell infiltrations suggest that IL-18 plays an important role in *H. pylori*-induced gastric inflammation^26^,^27^,^28^,^29^,^30^,^31^,^32^,^33^,^34^. Additionally, in *H. pylori*-infected patients, the CC genotype at the position −607A/C and GG genotype at the position −137G/C were associated with higher levels of IL-18 and severe gastric inflammation compared with other genotypes^33^,^34^. Therefore, *IL-18* gene polymorphisms may modulate the overall capacity of IL-18 production and could play important roles in host susceptibility and the outcome of *H. pylori* infection.

In our study, the GG genotype at position *−*137G/C, the TT genotype at position +113T/G, and the CC genotype at position +127C/T were more frequent in the *H. pylori*-positive group than in the *H. pylori*-negative group, indicating that these three SNPs might play a crucial role in *H. pylori* infection. Additionally, the C allele at position *−*137G/C, the G allele at position +113T/G, and the T allele at position +127C/T were less frequent in the *H. pylori*-positive group than in the *H. pylori*-negative group. However, these differences disappeared when adjusted for with Bonferroni correction, assuming 9 independent tests, except the C allele at position *−*137G/C. There were no significant differences between the *H. pylori*-negative and -positive groups at positions −656G/T and −607C/A.

One study^33^, regarding *IL-18* promoter polymorphisms at positions −137G/C and −607C/A, showed that in the Iranian population, the prevalence of the AA genotype and A allele at position −607C/A, but not at −137G/C, were significantly lower in *H. pylori*-infected duodenal ulcer patients than in *H. pylori*-negative subjects. This discrepancy might be attributed to a number of factors, including ethnic variability in *IL-18* genotype distribution, which differs among ethnic populations, sample size, and clinical heterogeneities, and the types of environmental factors involved in the pathogenesis of *H. pylori* infection.

The haplotype frequencies were used to determine the genetic correlation between these five SNPs, and the haplotype analysis showed that the haplotype CGT (−137C/ +113G/ +127T) was a protective haplotype, with higher frequency in the *H. pylori*-negative group than in the *H. pylori*-positive group. These results suggest that these three loci might have a combinatorial effect on *H. pylori* infection. Further functional research on these SNPs is warranted.

Our findings are not yet conclusive because it should be distinguished whether no significant difference is real or the sample size is too small to detect the differences. There are few *H. pylori*-negative subjects, especially in middle-aged or elderly hospital-based cohorts, because the prevalence of *H. pylori* in the Korean population is approximately 60% (36). We presented both non-adjusted OR and adjusted OR by age and sex. No replication data were included. Additionally, unfortunately, no additional data are available.

This is the first report that evaluated the association between the genetic variants in the *IL-18* gene and susceptibility to *H. pylori* infection in the Korean population with high prevalence. However, there are several limitations to our study. First, our study population was relatively small, which might influence some of the results, especially the lack of association between *H. pylori*-associated specific disease phenotypes. Second, we did not analyze the association between IL-18 production levels and *IL-18* gene polymorphisms. Third, our study focused only on the Korean population. Lastly, additional replication using an independent sample was not performed. Therefore, large-scale well-designed studies should be considered to confirm our results, avoid selection bias such as possible ethnic differences, and guard against the bias of repeated testing effects.

In summary, *IL-18* gene polymorphisms at positions −137G/C (rs187238), +113T/G (rs360718) and +127C/T (rs360717) exhibited an association with the susceptibility to *H. pylori* infection, before strict adjustment for multiple tests, in the Korean population. Furthermore, the haplotype CGT (−137C/ + 113G/ + 127T) was a protective haplotype with higher frequency in the *H. pylori*-negative group than in the *H. pylori*-positive group. The results indicate that the genetic variants in the *IL-18* gene may be associated with the susceptibility of *H. pylori* infection in the Korean population, suggesting that IL-18 plays a role in the pathogenesis of *H. pylori*-associated diseases.

## Patients and Methods

### Subjects

From January 2012 to September 2013, we recruited a total of 678 subjects in Korea who wished to receive a routine health check-up and a common screening examination. All participants were interviewed for their medical and family histories. Before the examination, the purpose of the study was explained to the participants, and informed consent was obtained from all individuals. This study was reviewed and approved by the Institutional Review Board of Chonnam National University Hwasun Hospital (Jeonnam, Korea), and written informed consent was obtained from all participating subjects. The present study was conducted according to the principles of the Declaration of Helsinki. All participants were Korean.

### Determination of *H. pylori* status

*H. pylori* infection status was determined using rapid urease testing (CLO test, Delta West, Bentley, Australia), and serum IgG, which is specific for *H. pylori*, was measured using an enzyme-linked immunosorbent assay (ELISA) (Genedia *H. pylori* ELISA; Green Cross Medical Science Corp, Eumsung, South Korea), with duplicate determinations according to the manufacturer’s guidelines. The Genedia kit used *H. pylori* antigen obtained from Korean *H. pylori* strains, with a sensitivity and specificity in Korean adults of 97.8% and 92.0%, respectively. The cutoff optical density (OD450 nm) of *H. pylori* IgG was 0.406. Two biopsy specimens were taken from the antrum and corpus during an endoscopic examination. These biopsy specimens were used for the CLO test. Blood samples were obtained from each participant. Isolated serum samples were neatly arranged in storage boxes and stored at −70 0C. If any one of these studies showed positive results, the patient was determined to be a current *H. pylori*-infected case. Only when all of the test results were negative was the patient considered to be negative.

### SNP search in database and primers

SNP sites in the human *IL-18* gene were obtained from the NCBI Genebank database (http://www.ncbi.nlm.nih.gov/projects/SNP/). We chose to focus on five SNPs [−656G/T (rs1946519), −607C/A (rs1946518), −137G/C (rs187238), +113T/G (rs360718) and +127C/T (rs360717)] of the promoter region in the human *IL-18* gene. SNP of the *IL-18* gene was determined by genomic polymerase chain reaction (PCR), and by direct sequencing. The primer pairs used in this study were as follows: for −656G/T and −607C/A forward 5’-GGTCAGTCTTTGCTATCA TTCCAGG-3’, reverse 5’-CCCCTTCCTCCCAAGCTCAAT-3’, for −137G/C, +113T/G and +127C/T forward 5’-AAGAGGTACAGGTTTTGGAAGGCA-3’, reverse 5’-TCCCGAAGCTGTGTAGACTGCA-3’.

### Genomic DNA extraction

Genomic DNA was extracted from peripheral blood mononuclear cells according to the manufacturer’s protocol by QIAamp DNA blood minikit (Qiagen, Valencia, CA). Briefly, blood samples with lysis buffer were incubated at 56°C for 10 min, and 100% ethanol of equal volume of the sample was added. The sample mixture was transferred to a QIAamp Mini spin column, and centrifuged at 6000*g* for 1 min. Flow-through containing lysis buffer was discarded, and DNA bound to the QIAamp column membrane was washed with wash-buffers. Genomic DNA was eluted from the QIAamp column membrane, using an elution buffer. The extracted DNA was stored at 4°C until analyzed.

### Direct Sequencing

PCR amplification was performed using 10 pmol of specific primer, 250 μM dNTPs, 50 ng of genomic DNA, and 1 U GoTaq Polymerase (Promega, Madison, WI) in the buffer provided by the manufacturer. The cycling conditions were 95°C for 20 s, 59°C for 20 s, and 72°C for 20 s. The PCR products were desalted and purified using a PCR product purification kit. The cleaned PCR products were analyzed using a Big Dye Terminator V3.1 Cycle Sequencing Kit (Applied Biosystems, Foster City, CA) following the manufacturer’s instructions. After ethanol purification, the reaction products were analyzed using a sequencer (ABI 3100, Life Technologies, Foster City, California, USA). SNP validation was visually confirmed.

### Statistical analysis

The data were analyzed using Statistical Package for the Social Sciences (version 15.0; SPSS, Chicago). The data are presented as the mean ± standard deviation (SD). The Chi-square test was used to compare genotype and allele frequencies between the *H. pylori*-negative and -positive groups, and Hardy-Weinberg equilibrium for any of the SNPs under consideration. The statistical significance of the difference between the *H. pylori*-negative and -positive groups was estimated by logistic regression analysis. Unadjusted and age- and sex-adjusted odds ratios were estimated. The haplotype frequencies were generated by HAPSTAT software (version 3.0), which simultaneously estimated the odds ratios (OR) parameters and haplotype distribution for five SNPs. Rare haplotypes with a frequency of less than 0.002 were excluded from analyses. The genotype allele specific risks and haplotype frequencies in the *H. pylori*-negative and -positive groups were estimated as OR and 95% confidence intervals (CI). The significance levels were corrected with the Bonferroni method in multiple comparisons (P = 0.05/9 = 0.0056), and a P value lower than the significance level was considered statistically significant in the analysis.

Our study subjects consisted of a homogenous population of Korean descent. We think no additional stratification was required.

## Additional Information

**How to cite this article**: Myung, D.-S. *et al*. Association between *interleukin-18* gene polymorphism and *Helicobacter pylori* infection in the Korean population. *Sci. Rep*. **5**, 11535; doi: 10.1038/srep11535 (2015).

## Figures and Tables

**Table 1:** Demographic and clinical characteristics of the study population.

Variables	Mean ± SD
Age, years	53.0 ± 10.8
BMI, kg/m^2^	24.1 ± 3.0
Variables	N (%)
Sex (male : female)	402:276 (59.3:40.7)
*H. pylori* status (negative : positive)	222:456 (32.7:67.3)
Smoking (never : current : previous)	384:137:157 (56.6:20.2:23.2)
Alcohol (never : current : previous)	243:394:41 (35.8:58.1:6.0)
Education (below : above college)	232:446 (34.2:65.8)
Income level $/month ( ≤ 2500 : 2500–8500 : ≥ 8500)	293:269: 116 (43.2:39.7:17.1)
Family history of gastric cancer (no : yes)	586:92 (86.4:13.6)

BMI, body mass index; *H. pylori, Helicobacter pylori*; SD, standard deviation.

**Table 2:** Demographic and clinical characteristics of the *Helicobacter pylori-* negative and -positive groups.

Variables	*H. pylori*(−) (N = 222)	*H. pylori*(+) (N = 456)	P value
Age, years	52.7 ± 10.9	53.2 ± 10.7	0.585
BMI, kg/m^2^	24.1 ± 3.3	24.1 ± 2.9	0.976
Male (%)	124 (55.9)	278 (61.0)	0.204
Alcohol consumption			
Never	81 (36.5)	162 (35.5)	0.812
Current	126 (56.8)	268 (58.8)	
Previous	15 (6.8)	26 (5.7)	
Smoking			
Non-smoker	131 (59.0)	253 (55.5)	0.375
Current smoker	38 (17.1)	99 (21.7)	
Ex-smoker	53 (23.9)	104 (22.8)	
Education			
≤12 years	69 (31.1)	163 (35.7)	0.230
>12 years	153 (68.9)	293 (64.3)	
Income ($/month)			
2500	99 (44.6)	194 (42.5)	0.858
2500–8500	85 (38.3)	184 (40.4)	
8500	38 (17.1)	78 (17.1)	
Family history of gastric cancer			
No	194 (87.4)	392 (86.0)	0.612
Yes	28 (12.6)	64 (14.0)	

BMI, Body mass index; *H. pylori, Helicobacter pylori*.

**Table 3:** Genotype and allele frequencies of *IL-18* gene polymorphisms in the *Helicobacter pylori-* negative and -positive groups.

Polymorphism	*H. pylori*(−) (N = 222)	*H. pylori*(+) (N = 456)	OR (95% CI)	P value	aOR* (95% CI)	P value
−656						
TT	65 (29.3)	130 (28.5)	1.00		1.00	
TG	114 (51.4)	220 (48.2)	0.97 (0.67–1.40)	0.851	0.97 (0.67–1.41)	0.873
GG	43 (19.4)	106 (23.2)	1.23 (.078–1.96)	0.376	1.25 (0.79–1.97)	0.349
Allele						
T	244 (55.0)	480 (52.6)	1.00		1.00	
G	200 (45.0)	432 (47.4)	1.10 (0.87–1.38)	0.421	1.11 (0.88–1.39)	0.392
−607						
AA	66 (29.7)	128 (28.1)	1.00		1.00	
AC	113 (50.9)	222 (48.7)	1.01 (0.70–1.47)	0.946	1.02 (0.70–1.48)	0.931
CC	43 (19.4)	106 (23.2)	1.27 (0.80–2.02)	0.309	1.29 (0.81–2.04)	0.288
Allele						
A	245 (55.2)	478 (52.4)	1.00		1.00	
C	199 (44.8)	434 (47.6)	1.12 (0.89–1.40)	0.338	1.12 (0.90–1.41)	0.315
−137						
GG	145 (65.3)	343 (75.2)	1.00		1.00	
GC	71 (32.0)	108 (23.7)	0.64 (0.45–0.92)	0.015	0.64 (0.45–0.91)	0.014
CC	6 (2.7)	5 (1.1)	0.35 (0.11–1.17)	0.089	0.35 (0.10–1.16)	0.085
Allele						
G	361 (80.3)	794 (87.1)	1.00		1.00	
C	83 (18.7)	118 (12.9)	0.65 (0.48–0.88)	0.005	0.64 (0.47–0.87)	0.005
+113						
TT	153 (68.9)	353 (77.4)	1.00		1.00	
TG	63 (28.4)	98 (21.5)	0.67 (0.47–0.98)	0.036	0.67 (0.46–0.97)	0.032
GG	6 (2.7)	5 (1.1)	0.36 (0.11–1.20)	0.097	0.36 (0.11–1.19)	0.093
Allele						
T	369 (83.1)	804 (88.2)	1.00		1.00	
G	75 (16.9)	108 (11.8)	0.66 (0.48–0.90)	0.011	0.66 (0.48–0.90)	0.010
+127						
CC	153 (68.9)	353 (77.4)	1.00		1.00	
TC	63 (28.4)	97 (21.3)	0.67 (0.46–0.97)	0.032	0.66 (0.46–0.96)	0.028
TT	6 (2.7)	6 (1.3)	0.43 (0.14–1.37)	0.153	0.42 (0.13–1.34)	0.143
Allele						
C	369 (83.1)	803 (88.0)	1.00		1.00	
T	75 (16.9)	109 (12.0)	0.67 (0.49–0.92)	0.013	0.66 (0.48–0.91)	0.011

*H. pylori, Helicobacter pylori;* OR, odds ratio; CI, confidence interval; aOR*, adjusted for age and sex.

**Table 4:** Estimated haplotype frequencies of IL-18 gene polymorphisms in the *Helicobacter pylori-* negative and -positive groups.

Haplotype −137/ + 113/ + 127	*H. pylori*(−) N (%)	*H. pylori*(+) N (%)	aOR* (95% CI)	P value
GTC	180 (81.1)	396 (86.8)	1	
CTC	4 (2.0)	5 (1.1)	0.63 (0.25–1.57)	0.319
CGT	37 (16.7)	53 (11.6)	0.64 (0.46–0.88)	0.006

All frequencies < 0.002 were ignored in the analysis. *H. pylori, Helicobacter pylori;* OR, odds ratio; CI, confidence interval; aOR*, adjusted for age and sex.
